# Genomic Selection for Ascochyta Blight Resistance in Pea

**DOI:** 10.3389/fpls.2018.01878

**Published:** 2018-12-20

**Authors:** Margaret A. Carpenter, David S. Goulden, Carmel J. Woods, Susan J. Thomson, Fernand Kenel, Tonya J. Frew, Rebecca D. Cooper, Gail M. Timmerman-Vaughan

**Affiliations:** The New Zealand Institute for Plant & Food Research Limited, Christchurch, New Zealand

**Keywords:** genomic selection, ascochyta blight, pea, disease resistance, genotyping-by-sequencing

## Abstract

Genomic selection (GS) is a breeding tool, which is rapidly gaining popularity for plant breeding, particularly for traits that are difficult to measure. One such trait is ascochyta blight resistance in pea (*Pisum sativum* L.), which is difficult to assay because it is strongly influenced by the environment and depends on the natural occurrence of multiple pathogens. Here we report a study of the efficacy of GS for predicting ascochyta blight resistance in pea, as represented by ascochyta blight disease score (ASC), and using nucleotide polymorphism data acquired through genotyping-by-sequencing. The effects on prediction accuracy of different GS models and different thresholds for missing genotypic data (which modified the number of single nucleotide polymorphisms used in the analysis) were compared using cross-validation. Additionally, the inclusion of marker × environment interactions in a genomic best linear unbiased prediction (GBLUP) model was evaluated. Finally, different ways of combining trait data from two field trials using bivariate, spatial, and single-stage analyses were compared to results obtained using a mean value. The best prediction accuracy achieved for ASC was 0.56, obtained using GBLUP analysis with a mean value for ASC and data quality threshold of 70% (i.e., missing SNP data in <30% of lines). GBLUP and Bayesian Reproducing kernel Hilbert spaces regression (RKHS) performed slightly better than the other models trialed, whereas different missing data thresholds made minimal differences to prediction accuracy. The prediction accuracies of individual, randomly selected, testing/training partitions were highly variable, highlighting the effect that the choice of training population has on prediction accuracy. The inclusion of marker × environment interactions did not increase the prediction accuracy for lines which had not been phenotyped, but did improve the results of prediction across environments. GS is potentially useful for pea breeding programs pursuing ascochyta blight resistance, both for predicting breeding values for lines that have not been phenotyped, and for providing enhanced estimated breeding values for lines for which trait data is available.

## Introduction

The pea, *Pisum sativum* L., is an important cool-season legume crop grown in temperate climates, with a global annual production of 17 million tons (FAOSTAT, 2014). It is nutritionally valuable, providing a rich source of protein and starch, as well as a range of other nutrients. The symbiotic relationship with nitrogen-fixing bacteria makes the pea an important component of sustainable cropping systems (Tayeh et al., 2015a). Pea cultivars are inbred lines which are largely homozygous, produced by several generations of self-fertilization after the initial cross. Therefore, it takes several years to produce genetically and phenotypically stable lines, and then to produce enough seed for field trials and commercialization. Increasing the efficiency of the breeding process by detecting elite lines earlier, using high-throughput genotyping or phenotyping, would be valuable.

Resistance to disease is an important target for pea breeding, and progress has been made using marker assisted selection (MAS) for pea diseases where resistance is a single gene trait. Polymerase chain reaction (PCR) markers are used to identify breeding lines carrying DNA polymorphisms closely linked to favorable alleles for selecting resistance to *Pea seed borne mosaic virus* (*sbm-1* and *sbm-2*; Gao et al., 2004), powdery mildew (*er-1*; Ghafoor and Mcphee, 2011; Pavan et al., 2011), and *Pea enation mosaic virus* (*En*; Jain et al., 2013). Several diseases remain problematic for the industry, particularly those in which disease resistance is multi-genic, e.g., Aphanomyces root rot (Desgroux et al., 2016) and ascochyta blight (Timmerman-Vaughan et al., 2002). Ascochyta blight typically reduces pea yield in an infected crop by 10%, or up to 60% if conditions favor it, and decreases the crop quality, so has a substantial effect on the industry (Liu et al., 2016). The ascochyta blight disease complex can involve three fungal pathogens: *Didymella pinodes* (Berk. & Bloxham) Verstergren, *Phoma medicaginis* Malbr. & Roum. var. *pinodella* (L.K. Jones) Boerema, and *Ascochyta pisi* Lib. Resistance to ascochyta blight is multi-genic, and numerous QTL associated with it have been discovered (Timmerman-Vaughan et al., 2002, 2004; Jha et al., 2014). Some studies have focused on susceptibility to *D. pinodes* (Tar’an et al., 2003; Prioul et al., 2004; Fondevilla et al., 2011a), where a number of associated QTL have also been identified. Microarray analysis has shown that hundreds of genes are differentially expressed during a resistant reaction to *D. pinodes* (Fondevilla et al., 2011b). Complete resistance to ascochyta blight has not been identified in pea, and measuring partial resistance is challenging because the disease prevalence in the field is influenced greatly by environmental factors such as rainfall, temperature and inoculum levels (Kraft et al., 1998). GS, which uses a large number of genetic markers located throughout the genome, may provide a solution for selecting elite breeding lines for a trait such as resistance to ascochyta blight which is multigenic and difficult to phenotype.

Genomic selection, which was initiated in the livestock industry, is now being used in a range of plant species, including fruit and timber trees (Kumar et al., 2011; Resende et al., 2012), and field crops such as wheat (Lopez-Cruz et al., 2015; Crossa et al., 2016), maize (Massman et al., 2013), and rice (Spindel et al., 2015). GS can enable selection to occur many years earlier in perennial plants where traits cannot be measured until later, e.g., fruit quality in apples (Kumar et al., 2011) and timber quality in pines (Resende et al., 2012). GS can shorten the breeding cycle for inbred annual crops such as wheat by allowing selection of new breeding parents, based on GEBV, to occur at an earlier filial generation (Bassi et al., 2016). GS has been shown to be an effective tool for increasing genetic gain per unit time for grain yield in maize (Massman et al., 2013; Beyene et al., 2015). GS studies in wheat have focused on the importance of genotype by environment interactions (Burgueño et al., 2012; Lopez-Cruz et al., 2015; Crossa et al., 2016) as a means of increasing prediction accuracy, both for predicting GEBV of lines that have not been phenotyped, and predicting GEBV across environments, for lines which have been phenotyped in some environments but not others. The way in which GS is used for breeding will vary greatly between crops, depending on genetics of the crop and the breeding and growing systems (Jonas and De Koning, 2013).

Genomic selection has been tested in pea for traits (thousand seed weight, number of seeds per plant, and flowering date) which are relatively easy to measure and highly heritable, using a SNP array for genotyping (Tayeh et al., 2015b). Cross-environment prediction accuracies were high (up to 0.83) for thousand seed weight. The authors concluded that GS was very promising for pea breeding provided that training and testing populations were chosen carefully, as the size and composition of the training population were found to have substantial influence on prediction accuracy. In a more recent study, GS was used to predict pea grain yield under drought conditions using GBS data (Annicchiarico et al., 2017). Genomic prediction of pea grain yield using GBS was accurate (with intra-population prediction accuracies for grain yield reaching 0.84 and inter-population 0.71) and cost-effective (phenotyping costs were estimated to be twice as much as genotyping costs). These results indicate that GS may be a valuable addition to pea breeding programs, however, its effectiveness for a retractable trait in pea is yet to be determined.

Genomic selection analysis is a two-step process: first genotypic and phenotypic data from a training population are fitted to a model. The model can then be used as a tool to predict phenotypes in a testing population which has been genotyped but not phenotyped (Jonas and De Koning, 2013; Desta and Ortiz, 2014). Subsequent phenotyping of the testing population can be used to assess the accuracy of the prediction, and also to update the model. A range of models have been developed for GS analyses (Desta and Ortiz, 2014), with the goal of improving the prediction accuracy. Initially, Bayesian models BayesA and BayesB were compared to best linear unbiased predictors (BLUP) and least squares (LS) (Meuwissen et al., 2001), and later other Bayesian models were developed (Park and Casella, 2008; Habier et al., 2011). Other approaches include kernel regression, random forests, and neural networks (Gianola and Van Kaam, 2008; Desta and Ortiz, 2014). Comparisons of the various methods used for genomic prediction have not revealed a single model which is superior in all cases (Habier et al., 2011; Heffner et al., 2011) because model performance depends on the number of genomic regions influencing a trait and the size of the effects, and different models make assumptions that may or may not match the genetic architecture of the trait(s) of interest.

Genomic selection requires genotypic data from a large number of markers throughout the genome. These can be generated using SNP arrays or GBS approaches, such as whole genome resequencing or reduced representation sequencing (reviewed by Scheben et al., 2017). The reduced representation GBS method developed at Cornell University (Elshire et al., 2011), is a high-throughput method, effective for generating single nucleotide polymorphism (SNP) data from a large number of DNA samples, and consequently has been used in numerous GS studies (Bhat et al., 2016). This method produces sequences from regions adjacent to restriction enzyme sites, and by using methylation-sensitive restriction enzymes, can target coding regions rather than repetitive DNA. This makes it an ideal method for large genomes with abundant repetitive sequences, such as pea.

Genomic selection analyses commonly report prediction accuracy based on the correlation between predicted and observed trait values. However, in addition to estimates of breeding value (BV) for the whole population, plant breeders often want an accurate prediction of the top individuals to be selected as elite cultivars or parents for the next round of breeding (Bassi et al., 2016). GS has the potential to contribute to pea breeding in two ways: by enabling the prediction of GEBV in unphenotyped individuals, and by improving the accuracy of the BV of individuals that have been phenotyped, particularly for recalcitrant/hard-to-measure traits, by combining trait data from multiple environments and/or years with genotypic data. The use of molecular data to estimate BV is fundamentally more accurate than the use of pedigree data (Hayes et al., 2009), because molecular data generate a realized relationship matrix, whereas a pedigree-based matrix uses expected values in which siblings all have an average and equal value.

Here we report the evaluation of GS for breeding ascochyta blight disease resistance in a pea breeding program. GBS was used to genotype the training population. We compared the ability of several GS models to predict phenotypes, and explored the effects SNP quality and number, spatial analysis of phenotype data, and single- versus two-stage analyses, on the prediction accuracy.

## Materials and Methods

### Plant Material

The training population was a collection of 215 lines from PFR pea breeding program made up of current PFR breeding lines and commercial cultivars from both PFR and elsewhere, some of which have been used as parents in the PFR breeding program (Supplementary File 1). Some of these lines have been bred for ascochyta blight resistance.

### Phenotyping

Ascochyta blight resistance was estimated for the training population as a disease severity score (ASC). ASC was determined from two field trials located in Gore, Southland, New Zealand (46.11 S, 168.89 E); one sown in 2013 and one in 2015. The trial site is subject to naturally occurring field epidemics of ascochyta blight and the pathogens at this site have previously been characterized as *D. pinodes* and *P. medicaginis* (Timmerman-Vaughan et al., 2002, 2016). The trials were sown in early November and scored in February of the following year. Each trial comprised three biological replicates and was set out as a randomized complete block design with three blocks; each block contained 3 rows and 77 columns. One breeding line (“Ashton,” Seminis) was used as a control and planted in every 11th plot. Each plot was sown with 50 seeds in a single row. Management of the trials included herbicide application but no irrigation, fertilizer or fungicide. Ascochyta blight disease was allowed to develop spontaneously from environmental sources. Each plot was given a score based on visual appearance of the whole plot using a scale from 1 to 5 with increments of 0.5. Scores were: 1 = nearly symptomless plants, 2 = trace of infection on stem and leaves, pods clear of infection, 3, leaves strongly affected, slight penetration of the stem, and less than 25% of the pod surface covered by lesions, 4, leaves strongly affected, complete girdling of the stems and 25–50% of the pod surface covered by lesions, and 5, all plants severely infected, lesions coalesced, vine blue black, and >50% of the pod surfaces covered by lesions. The trials were scored when most plots were at the pod fill stage, a time when disease severity between lines was readily distinguished.

### Spatial Analysis of Phenotypic Data

To adjust for possible spatial effects in the ascochyta blight field trials, a linear mixed effects model was fitted to the 2 years of data using ASReml-R (Butler et al., 2009). A model with year as a fixed effect, variety and row as random effects, and a first-order separable autoregressive (AR1xAR1) variance model (with both the row effect and the AR1xAR1 variance structure allowed to vary by year) was determined to be an appropriate model, following the method of Gilmour et al. (1997), and was significantly better than a base model with year as a fixed effect and variety as a random effect (*p* < 0.001). The final model gave a BLUP for each variety which was then used in GS analyses (ASC Spatial). Simple (unadjusted) means were also calculated across the three plots for each year (ASC2013 and ASC2015), and across both years (ASC Mean), and used in the GS models.

### Genotyping

DNA was purified from young pea leaf samples using a DNeasy Plant Mini Kit (Qiagen, Hilde, Germany) and quantified using a Quant-iT PicoGreen dsDNA Assay Kit (Thermo Fisher Scientific, MA, United States). GBS libraries were constructed according to the method of Elshire et al. (2011) with some modifications as detailed below. Sets of 48 barcoded adaptors were designed by Deena Bioinformatics^1^, and supplied as BioRP-purified oligonucleotides by Bioneer Corporation (Daejeon, Korea). Adaptor oligonucleotides were dissolved at 50 μM, then equal volumes of plus and minus strands were mixed and annealed by incubation at 95°C for 2 min, ramped down to 25°C by 0.1°C/s, and incubated at 25° C for 30 min. Annealed adaptors were diluted 1/100 in water and the concentration determined using Picogreen. Barcoded and common adaptors were combined so that each adaptor had a concentration of 0.3 ng/μl. The PCR primers were PAGE (polyacrylamide gel electrophoresis) purified from Integrated DNA Technologies (Coralville, IA, United States). Adaptor, barcode and primer sequences are listed in Supplementary File 2. DNA (200 ng) was digested with 5 U *Ape*KI enzyme (NEB, Ipswich, MA, United States) at 75°C for 2 h in NEB buffer 3. Adaptors were ligated to the digested DNA by adding 0.9 ng of each of the barcoded and common adaptors with 400 U T4 DNA ligase (NEB) in ligase buffer, the amount of adaptor being previously determined by titration (Elshire et al., 2011). Ligation reactions were incubated at 22°C for 2 h then inactivated at 65°C for 30 min. Ligation products were purified using Ampclean magnetic beads (MAGBIO genomics, Gaithersburg, MD, United States), then amplified by PCR using Taq 2× mastermix (NEB), with 25 pmol of each primer, and 10 μl ligation products in 50 μl, in individual reactions. The PCR was initiated at 72°C for 5 min, followed by 98°C for 30 s, then 18 cycles of 98°C for 10 s, 65°C for 30 s, 72°C for 30 s, finishing with 72°C for 5 min. Amplified libraries were checked by electrophoresis in an agarose gel to ensure they had successfully amplified. Aliquots of 10 μl per amplified library were pooled, in batches of 48, then purified using Ampclean magnetic beads and eluted into 40 μl. The pooled libraries were sequenced using the Illumina HiSeq 2000 platform at the Australian Genome Research Facility (Brisbane, Australia), using one lane per pooled library batch of 48 samples. The sequencing generated 100 bp single-end reads, reading from the end of each construct that contained the internal barcode.

Raw data was assessed using FastQC (Andrews, 2010) for sequence quality and presence of adapter read through. Sequence data was also assessed by barcode splitting and enzyme integrity checking using the ea-utils package, “fastq-multx” (Aronesty, 2011). The UNEAK (Universal Network Enabled Analysis Kit) pipeline (Lu et al., 2013), which does not use a reference genome sequence, was used to process the raw sequence reads and identify SNPs by aligning similar reads to each other UNEAK was run using default conditions, with the minimum number of reads (–c) set to 5. The resulting hapmap files were imported into TASSEL v3.0.165 (Bradbury et al., 2007) for filtering on SNP and taxa quality. Data from unwanted lines (negative controls, duplicates, any with inadequate data) were removed. Heterozygous base calls were coded as missing data because inbred pea lines are expected to be almost entirely homozygous, so heterozygous base calls are likely to be errors. Any SNPs with a minor allele frequency of <0.05 were removed. SNPs were filtered using three thresholds for missing data: ≥50, 70, and 90% of lines have SNP data (<50, 30, and 10% missing data, respectively). The subsequent analyses made use of the ≥90% dataset, with the exception of a comparison between the different thresholds.

Alleles were recoded as the number of copies of the minor allele (0 or 2), using the R package “synbreed” (Wimmer et al., 2012). Missing data was imputed as “0,” representing the major allele, as in the absence of a pea genomic sequence, the order of SNPs was not known so imputation based on haplotypes was not possible. A principle component analysis (PCA) of the genotypic data was conducted using the R package “stats.” Population structure was determined from the genotypic data using the program STRUCTURE v2.3 (Pritchard et al., 2000) and the best estimate of K (the number of subpopulations) was determined by the method of Evanno et al. (2005).

### Genomic Selection Analyses

The performance of different GS models at predicting BV of unphenotyped individuals was compared using the R package “BGLR” (Bayesian Generalized Linear Regression) (Perez and De Los Campos, 2014) to run GBLUP, BayesA, BayesB, BayesC, BRR, BL, and RKHS analyses. SNP data quality thresholds of 90, 70, and 50% (i.e., % of lines that have data for a SNP) were compared using the “BGLR” package to run GBLUP and BayesA models. Both analyses comprised 500 random partitions consisting of a training set of 150 individuals and a testing set of 50, from the 200 lines for which there were SNP and trait data. The trait data used were ASC Mean, ASC Spatial, and the means from the individual ascochyta blight trials, ASC2013 and ASC2015. GS analysis incorporating marker × environment (M × E) interactions was conducted for ASC2013 and ASC2015, using “BGLR” with a GBLUP model (Lopez-Cruz et al., 2015) with 500 random partitions and a testing set of 50. Three models were compared: a single environment model in which there is no borrowing of information across environments; an across environment model where information is borrowed across environments but marker effects are constant across environments; and a M × E interaction model where information is borrowed across environments and marker effects are allowed to change across environments. Two M × E cross-validation analyses were done: one for predicting GEBV for individuals which had not been phenotyped (CV1) as in the previous analyses, and another (CV2) for predicting GEBV for individuals in an environment in which they had not been phenotyped, based on results from phenotyping in another environment. Finally, the accuracy of GS analysis using phenotype data from a single trial to predict GEBV of individuals in a second trial was determined, using a GBLUP model with “BGLR.”

Using ASReml-R, a GBLUP cross-validation was carried out to compare a single-stage approach to five different non-weighted two-stage approaches. In the two-stage analyses, phenotypic means (or adjusted means) were calculated first, then used in the GS analysis, whereas in the single-stage analysis the spatial effects, multiple environments and genomic data were combined in a single model to predict GEBVs. The five two-stage approaches were: using the 2013 and 2015 ASC means separately (referred to as 2013 and 2015, respectively); combining the 2 years’ data as a grand mean (Mean); combining the 2 years’ data via a bivariate analysis (Bivariate); and combining the 2 years’ data via a spatial analysis to give an adjusted mean (Spatial); (Table 1). The genomic relationship matrix (GRM) was calculated using the R package “cpgen” (Van Raden, 2008), with lambda = 0.01. For each of 500 iterations, a test set of size 50 out of the 215 breeding lines was randomly selected; the training set (165 lines) was used to fit the five different two-stage, and the single-stage, models; and, finally, the correlation between predicted and observed ASC score of each test set was calculated for each model.

**Table 1:** Summary of the two-stage and single-stage genomic selection analyses.

Two-stage models tested for genomic selection		
**Model name**	**Stage 1: phenotypic value calculated for use in Stage 2**	**Stage 2: model fitting, phenotypic value and genotypic data**	**Observed values (used for comparison to predicted values)**

2013	2013 means	LMM with GRM	2013 means
2015	2015 means	LMM with GRM	2015 means
Mean	Mean of 2013 and 2015 means	LMM with GRM	Mean of 2013 and 2015 means
Bivariate	2013 means and 2015 means	Bivariate LMM with GRM	Mean of 2013 and 2015 means
Spatial	Adjusted mean (BLUP) LMM: (2013 means, 2015 means) ∼ year (fixed) + variety, row (random) + spatial autocorrelation	LMM with GRM	Adjusted mean (BLUP)

**Single-stage model tested for genomic selection**		

Single-stage	LMM: (2013 means, 2015 means) ∼ year (fixed) + variety, row (random) + spatial autocorrelation + GRM		Adjusted mean (BLUP)

*LMM, linear mixed model; GRM, genomic relationship matrix; BLUP, best unbiased linear predictor.*

## Results

### Genotypic Data

Sequencing of GBS libraries generated 1.9–5.7 million reads per pea line, with a median read count of 3.9 million. This equated to an estimated 0.1× coverage of the genome (per sample) based on a genome size of 4.3 Gb. Analysis of GBS data using the UNEAK pipeline produced 74,738 SNPs, however, most of these SNPs had missing data for many pea lines. After the SNP data had been filtered to remove those with minor alleles with a frequency of <0.05, and those with excessive missing data and/or heterozygous base calls, the numbers of SNPs were as follows: 14,451 SNPs had data for ≥50% of pea lines; 8954 SNPs had data for ≥70% of pea lines, and 6019 SNPs had data for ≥90% of pea lines (Supplementary File 3). Of the 215 pea lines, there were 200 for which both SNP and trait data were available, so these were used in the following analyses.

**FIGURE 1:**
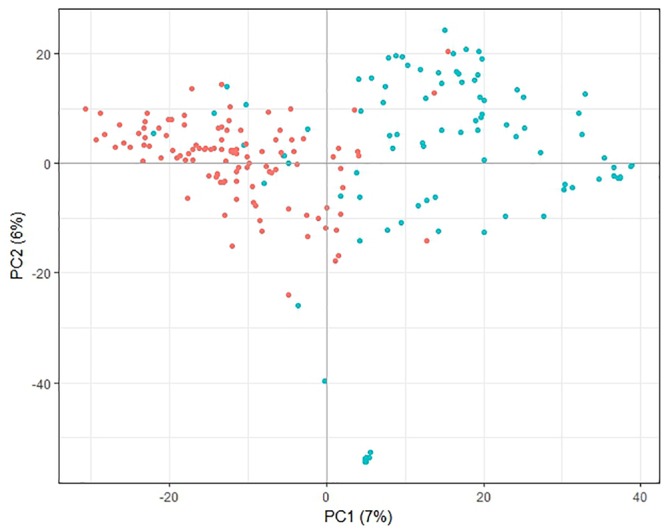
Principal component analysis plot of pea genotypic data. Lines originating from The New Zealand Institute for Plant and Food Research Limited (PFR) breeding program are shown in blue, lines from other sources are red. The percent variance associated with each of the first two principal components (PC) is shown in brackets.

Principle component analysis of the genotypic data showed that the first principle component separated most of the lines from the PFR breeding program from most of the lines from other sources, while the second principle component separated a small group of the PFR lines from the remaining lines (Figure 1). However, the first two principal components accounted for only 7 and 6% of the variation, respectively, therefore the trends described above are weak. Analysis of population structure also confirmed that there was little structure in the population based on the shape of the LnP(D) curve, and that the population structure was best represented by 2 or 10 sub-populations (Supplementary File 4).

### Phenotypic Data

**FIGURE 2:**
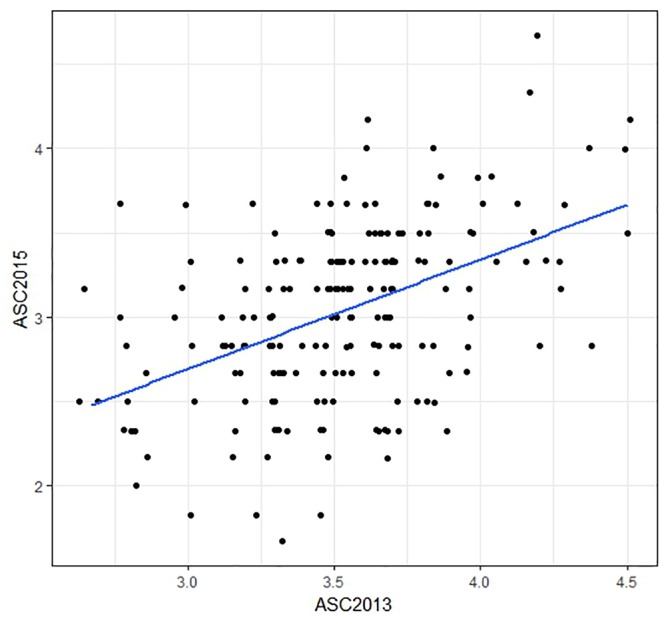
Scatter plot comparing ascochyta blight disease scores (ASC) from two field trials. The plotted values are means of three biological replicates, with a regression line shown in blue. The correlation between the data from the two field trials was *r* = 0.46. Points which are over-plotted have been made visible using the jitter function in R package “ggplot2.”

The ASC trait showed greater variation in the 2015 field trial (range 1.7–4.7, mean 3.0) than in 2013 (range 2.7–4.5, mean 3.5). Histograms of trait data are provided in Supplementary File 5. Correlation (Pearson) between the mean ASC scores from the two field trials was only moderate (*r* = 0.46, Figure 2). The spatial analysis identified field trends with row and proximity effects (for both years), whereas column effects were negligible. The mean values and the spatially adjusted means for ASC were highly correlated (0.96) and spatial adjustment produced little change in the ranking of the lines.

### Comparison of Different GS Models

The genotypic and phenotypic data were analyzed to determine the extent to which the prediction accuracy was affected by the choice of GS model, and by the SNP quality and number (i.e., fewer SNPs with less missing data versus more SNPs with a higher proportion of missing data). Next, different ways of combining the ASC data from two field trials for a two-stage GBLUP analysis were explored (mean, spatially adjusted mean, and bivariate) and compared to a single-stage analysis. Finally, the breeding lines were ranked with respect to ASC using the two-stage and single-stage GBLUP analyses, to determine how consistently the methods predicted the top ten lines.

**FIGURE 3:**
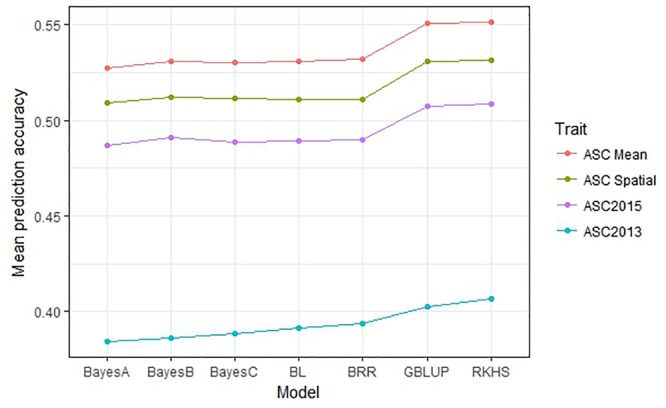
Mean prediction accuracy for seven genomic selection models calculated from 500 randomized testing/training partitions. Prediction accuracy is the correlation between predicted and observed trait values for the testing set.

**FIGURE 4:**
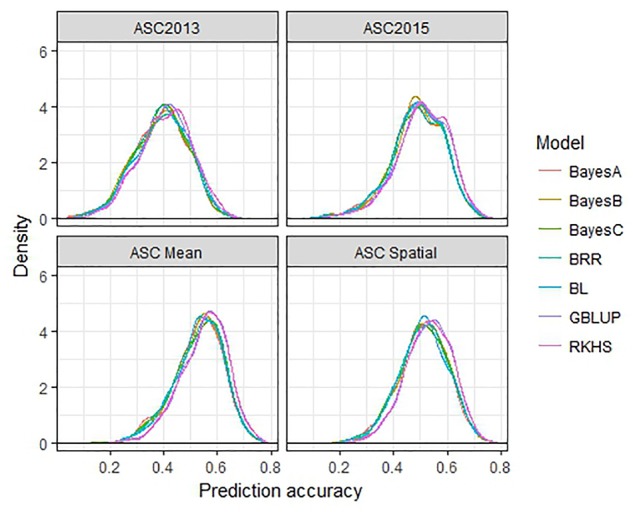
Density plots of prediction accuracy for seven genomic selection models using 500 randomized testing/training partitions.

The performance of seven GS models (GBLUP, BayesA, BayesB, BayesC, BRR, BL, and RKHS) was compared using the trait values ASC2013, ASC2015, ASC Mean, and ASC Spatial (Figure 3). In all cases, GBLUP and RKHS gave slightly higher mean prediction accuracies than the other models (*p* < 0.01), but overall, the differences between the models’ prediction accuracies were small, ≤0.02. The mean prediction accuracy for ASC was greatest when the trait value used was the mean of the 2 years (Figure 3, ASC Mean, 0.55), which was slightly better than using the trait value based on spatial analysis (Figure 3, ASC Spatial). The prediction accuracies obtained from individual partitions of the 200 pea genotypes into testing and training sets fell over a large range for all traits (e.g., 0.2–0.8 for ASC Mean). Density plots of these accuracies for the seven models tested are presented in Figure 4. These plots indicate that the partitioning of the testing and training sets had a large effect on the prediction accuracy.

**FIGURE 5:**
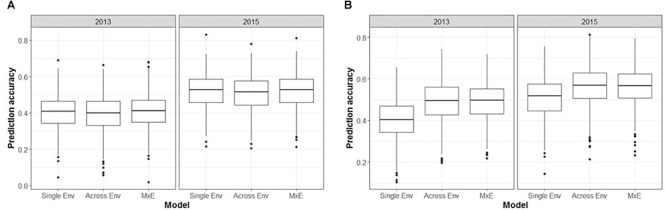
Box plots from GS analysis incorporating marker × environment (M × E) showing the distributions, median values, 25 and 75% quantiles, and outliers for the prediction accuracies obtained from 500 testing/training partitions, for ascochyta blight disease score from the 2013 and 2015 pea field trials. **(A)** Cross-validation of predicted breeding values for lines with no phenotypic data available (CV1). **(B)** Cross-validation of predicted breeding values for lines which had phenotype data available from one environment but not the other (CV2). Three models were compared: single environment, across environment, and M × E interaction.

The inclusion of M × E interactions made little difference to the prediction accuracies obtained for unphenotyped individuals (CV1, Figure 5A). The three models (single environment, across environment, and M × E interaction) generated mean prediction accuracies of 0.40, 0.39, and 0.41, respectively, for ASC2013, and 0.52, 0.51, and 0.52, respectively, for ASC2015. In both cases the across environment model, which assumes that effects are constant across environments, gave the lowest accuracy, but the differences were not significant (*p* > 0.09). However, prediction accuracies were greater when using phenotype data from one environment to predict phenotypes in a second environment (CV2, Figure 5B). The single environment, across environment, and M × E models produced mean prediction accuracies of 0.40, 0.49, and 0.49, respectively, for ASC2013, and 0.51, 0.56, and 0.56, respectively, for ASC2015. In both cases, the across environment and M × E models gave higher mean accuracy than the single environment model (*p* < 0.001).

**FIGURE 6:**
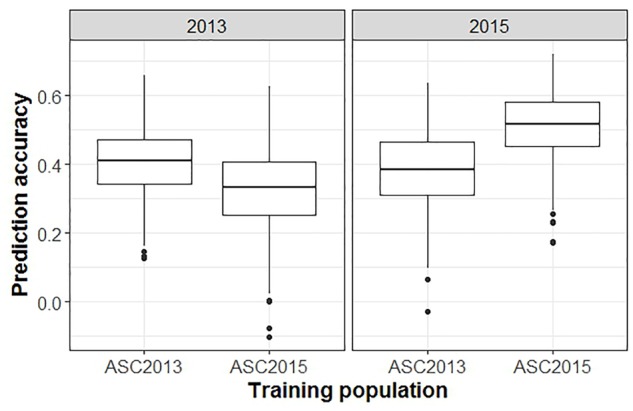
Box plots from a GBLUP GS analysis comparing accuracy of predictions across trials versus within trials. Plots show the distributions, median values, 25 and 75% quantiles, and outliers for the prediction accuracies obtained from 500 testing/training partitions, for ascochyta blight disease score from the 2013 and 2015 pea field trials. The panel on the right shows the accuracy when GEBV predicted from ASC2013 and ASC2015 training populations is compared to test populations from ASC2015.

When GEBVs of the testing set were based on phenotypic data from a single ascochyta blight field trial, and compared to the phenotypic values from a second trial, the mean prediction accuracy was notably lower than in the previous analyses (Figure 6, *p* < 0.001). GEBVs for ASC2015, based on ASC2013 phenotypic data, were generated with an accuracy of 0.38, whereas the converse gave 0.34.

### Comparison of SNP Quality Thresholds

**FIGURE 7:**
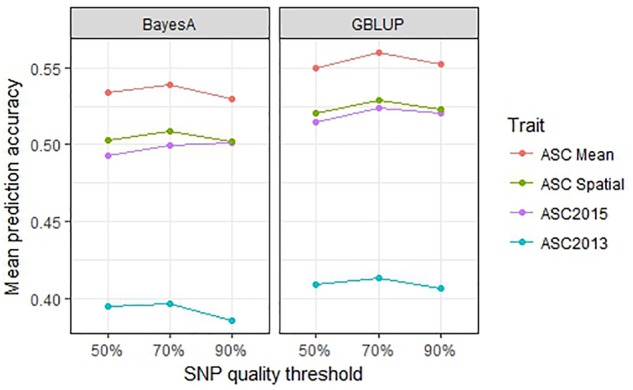
Mean prediction accuracy for genomic best linear unbiased prediction (GBLUP) and BayesA calculated from 500 testing/training partitions using three different single nucleotide polymorphism (SNP) quality (missing data) thresholds.

Genomic selection prediction accuracy was compared across SNP datasets filtered according to their amount of missing data to determine whether best results were obtained using fewer SNPs and minimal missing data, or more SNPs with a larger proportion of missing data. The 90% data set comprised 6019 SNPs for which data was missing for <10% of lines, with an average of 3% missing data overall. Similarly, the 70% dataset had 8954 SNPs each with missing data for <30% of lines, with an overall mean of 9% missing data. The 50% dataset had 14,451 SNPs with a mean of 21% missing data overall. The GBLUP and BayesA methods were used for these comparisons. For all trait values, the 70% SNP filtering threshold gave the highest mean prediction accuracies, however, the differences were minor (≤0.02, *p* > 0.1) for both GBLUP and BayesA analyses (Figure 7). Since the addition of SNPs of lower quality (i.e., with more missing data) did not notably improve the prediction results, while the smaller, higher quality 90% dataset had a reduced computational time (i.e., approximately half that of the 50% dataset), it appears practical to use the smaller dataset.

An additional comparison was made between imputing with the minor allele instead the major allele, using the 90% SNP dataset with GBLUP and BayesA. The imputation difference had a minimal effect on prediction accuracy (data not shown). Imputation using the major allele was estimated to be correct in 76% of cases, based on the frequencies of the major and minor alleles prior to imputation.

### Two-Stage Versus Single-Stage Analyses

**FIGURE 8:**
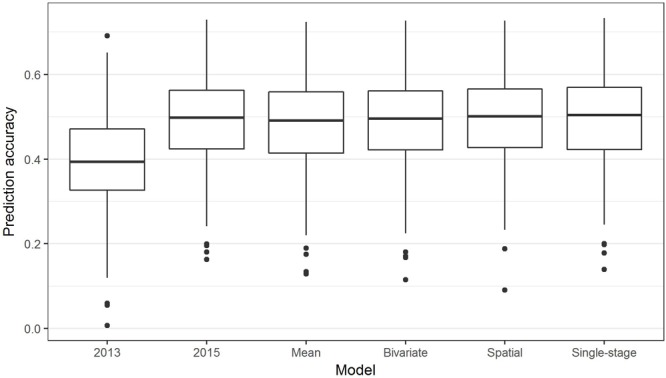
Box plots showing the distributions, median values, 25 and 75% quantiles, and outliers for the prediction accuracies obtained from various two-stage (2013, 2015, Mean, Bivariate, and Spatial) and single-stage approaches to breeding value estimation for ascochyta blight disease score (ASC), calculated from 500 testing/training partitions.

The above analyses using the R package BGLR were all non-weighted two-stage approaches to GS, in which phenotypic means were calculated in the first stage (for example, to adjust for spatial effects or multiple environments), then fed into a model to predict GEBVs based on SNPs. In contrast, in a single-stage approach, spatial effects and multiple environments are modeled at the same time as incorporating SNP information to predict GEBVs. Box plots of the prediction accuracies generated by various two-stage approaches and a single-stage analysis (Table 1) are shown in Figure 8, and indicate that the single-stage approach did not substantially improve the prediction accuracy of the test sets.

### Performance of Genomic Selection in Pea

The greatest mean prediction accuracy achieved for ASC was 0.56, obtained using GBLUP analysis with a mean value for ASC and data quality threshold of 70% (i.e., missing data in <30% of lines).

As the heritability of a trait may affect the accuracy of GS, heritabilities were estimated using the GBLUP model. The calculated heritabilities followed a similar trend to the prediction accuracies, i.e., 0.53 and 0.59 for ASC2013 and ASC2015, and 0.61 and 0.63 for ASC Spatial and ASC Mean.

### Selecting Elite Lines

Plant breeders are interested in identifying the elite lines in a breeding program, in addition to determining trait values for all lines. The predicted top 10 lines for ASC, as determined by the two-stage and single-stage methods using GBLUP, are presented in Table 2. Despite little difference in average prediction accuracy of the different methods, there was variation in the ranks for the top 10 breeding lines. However, estimates for the top three lines were consistent across the analyses which combined data from the two trials (Mean GBLUP, Bivariate GBLUP, Spatial GBLUP, and single-stage).

**Table 2:** Top 10 ranked lines for ascochyta blight resistance (ASC) according to the single-stage genomic best linear unbiased prediction (GBLUP) method, along with their rankings according to six other methods.

Variety	Single-stage GBLUP	Spatial GBLUP	Bivariate GBLUP	Mean GBLUP	2015 GBLUP	2013 GBLUP	Simple means (no genotype data)
PFR00-8212	1	2	1	1	1	7	1
S2-271	2	1	2	2	10	1	8
K95-2198	3	3	3	3	3	6	3
K95-2063	4	4	30	36	87	2	22
PFR92-845	5	17	4	18	2	76	3
PFR10-A24	6	6	16	19	30	16	11
Foli_1	7	8	7	8	6	31	15
Supergreen	8	14	5	15	36	5	6
PFR00-8264	9	7	8	4	8	11	2
PFR00-8257	10	5	11	5	13	12	8

## Discussion

Genotyping-by-sequencing (Elshire et al., 2011) proved to be an effective method for generating SNP data for GS in pea, with a simple and efficient laboratory protocol. The amplification of samples individually, rather than amplifying pooled samples, made the process less efficient but allowed us to ensure that all samples were successfully amplified. The use of magnetic beads for the clean-up steps gave results consistent with spin columns (data not shown) but required less handling time. Although GBS resulted in a large number of SNPs with missing data for many lines, the overall number of SNPs identified was so large that there were plenty that had little missing data (>6000 SNPs had data for ≥90% of samples). The results of other studies suggest that this number is quite sufficient for GS. Although the accuracy of GS increases with increased marker density, the effect is small (Heffner et al., 2011; Tayeh et al., 2015b) and in barley and wheat reached plateau at around 1000 and 1500 markers, respectively (Bonnett et al., 2013; Oakey et al., 2016). Similarly, the size of the training population influences prediction accuracy, with little increase in prediction accuracy between 210 and 240 individuals in pea (Tayeh et al., 2015b), suggesting that the number of individuals used in this study was adequate. The average genome coverage was low at approximately 0.1×, but because the sequence reads were generated only at *Ape*KI restriction sites, the subset of the genome which was sequenced would have had much higher read coverage.

The UNEAK analysis pipeline was designed to be used in any species, irrespective of the availability of a genome sequence, making it were ideal for a pea GS study. However, aligning to a genome would be preferable as the SNP positions would be known, allowing imputation of missing data based on haplotypes. Synteny between the *Medicago truncatula* and pea genomes (Aubert et al., 2006; Young et al., 2011) suggested that aligning the pea GBS reads to the *M. truncatula* genome might prove useful. This was attempted, as well as aligning to an unpublished fragmented pea genome. However, in both cases the number of SNPs generated was less than with UNEAK (data not shown). Finally, the pea GS SNPs were ordered using GBS SNPs from a pea mapping population, however, that also reduced the number of SNPs available due to the lower genetic diversity within the mapping population. Therefore, we chose to use the SNPs derived from the UNEAK analysis, and to stringently filter out SNPs that had excessive missing data, to provide optimal data for the GS analyses. Imputation with the major allele, which was estimated to be correct in 76% of cases, was considered to be a satisfactory alternative to imputation based on a genome sequence.

Analysis of the SNP data revealed little population structure among the lines used for GS. The PCA plot showed some separation of the lines bred at PFR from other lines, but the two groups overlapped. This is consistent with some of the other lines having been used as parents in the PFR breeding program. Strong population structure can decrease the cross-validation accuracy of GS (Habyarimana, 2016) so the weak population structure observed here is advantageous.

The choice of model used in the GS analysis made small but significant (*p* < 0.01) differences to the mean prediction accuracies obtained. GBLUP and RKHS (which both use a GRM rather than calculating marker effects) gave slightly higher prediction accuracies than the other models (Figure 3). Although the choice of model can sometimes make a difference, depending on the number of QTL which affect the trait (Lin et al., 2014), other studies have found that the choice of model does not affect the prediction accuracy substantially in pea (Tayeh et al., 2015b), wheat (Heffner et al., 2011), and dairy cattle (Habier et al., 2011). It was noted that the prediction accuracy of individual testing:training partitions was highly variable, which emphasizes that the choice of training population is critical and has a substantial effect on the ability to predict phenotypes. Previous studies have shown that prediction accuracies can be increased by optimizing the division of samples into testing and training sets so that the relatedness between individuals in the testing and training sets is maximized (Rincent et al., 2012; Tayeh et al., 2015b; Bassi et al., 2016).

The best prediction accuracy obtained in this study, for lines which had not been phenotyped, was 0.56, using a GBLUP model and the mean of two field trials. Previous GS studies of multi-genic traits in pea have achieved higher prediction accuracies than this, but have also used traits which have higher heritability and/or gave more consistent phenotypic data. A previous study in pea (Burstin et al., 2015) attained a prediction accuracy of 0.62 for the highly heritable trait thousand seed weight (*h*^2^ = 0.98) but lower accuracies for flowering time and number of seeds (0.46 and 0.39, respectively) which, compared to thousand seed weight, had lower heritability (*h*^2^ = 0.71 for number of seeds) or less consistency between trials (*R*^2^ = 0.62 for flowering time). Another pea study using the same trait data but with a SNP array to produce more markers (Tayeh et al., 2015b) gained higher prediction accuracies of 0.83, 0.68, and 0.65 for the three traits, respectively, all of which had higher heritability than the ASC mean reported here (*h*^2^ = 0.63). A more recent study (Annicchiarico et al., 2017) achieved prediction accuracies of 0.54–0.84 for highly heritable (*h*^2^ = 0.9) pea grain yield under drought stress, when predicting within bi-parental populations with limited genetic variation and a limited number of relevant QTL. However, the inter-population prediction abilities were much lower.

The ASC2015 trait consistently gave higher prediction accuracies than ASC2013, which is likely due to the greater spread of the disease scores in 2015 (Figure 2 and Supplementary File 2) which provided better discrimination between lines. The cross-environment prediction accuracies, where GEBVs calculated using phenotypic data from one field trial were compared to phenotypic data from a second trial, were relatively low (Figure 6). This is not unexpected given that the correlation between the two phenotypic data sets was only moderate (*r* = 0.46). The variation in the distribution of the ASC trait values, and the moderate correlation, between years are consistent with the extensive influence of environmental variables on the disease severity, including variation in the composition of natural pathogen populations in the field. Previous research revealed that ascochyta blight in pea was associated with the presence of both *D. pinodes* and *P. medicaginis* (Timmerman-Vaughan et al., 2016), so variation in the disease scores between years could be due to changes in strains or the relative amounts of these two pathogens. Consequently, when using GS for breeding peas with resistance to ascochyta blight, it may be advisable for the phenotypic values for the training population to be based on at least two field trials.

The inclusion of M × E interactions in a GBLUP GS model did not improve the prediction accuracy for lines which had not been phenotyped (CV1, Figure 5A). The results were consistent with previous results from wheat (Lopez-Cruz et al., 2015) in that the single environment and M × E models gave slightly higher prediction accuracy than the across environment model. However, for CV2, where phenotypic data from one environment were used to predict GEBV for a second environment, an increase in prediction accuracy was gained in the across environment and M × E models compared to the single environment model (Figure 5B). For comparison, the CV2 prediction accuracies for wheat were highest for the M × E model, and lowest for the across environment model (Lopez-Cruz et al., 2015), for most of the environments analyzed. In both pea and wheat, the CV2 M × E model gave greater prediction accuracy than CV1 M × E model, indicating that there may be value in an approach where different subsets of breeding lines are phenotyped in different environments, provided that the environments are similar enough to produce positively correlated phenotypic data (Lopez-Cruz et al., 2015).

The filtering threshold of the SNP data also made little difference to the prediction accuracy (Figure 7). Therefore it makes sense to remove SNPs that are poorly represented among the lines, and thereby reduce the computational time. Increasing the number of markers used in GS is known to improve prediction accuracies, but this is a small effect (Heffner et al., 2011) that reaches a plateau. A recent study with GBS data in pea (Annicchiarico et al., 2017) found that increasing the missing data threshold from 10 to 50% of lines caused a substantial increase in the number of markers, thereby improving the predictive ability. However, the increase in predictive accuracy plateaued; increasing the number of markers up to 400–500 improved prediction accuracy, but further increases produced little gain. In this case, bi-parental populations with relatively narrow genetic variation were used, which explains why a small number of markers was sufficient for GS. Similarly, in a large and diverse cohort of wheat breeding lines, increased markers produced an increase in predictive ability that reached a plateau at approximately 1500 markers (Bonnett et al., 2013). Therefore, it is likely that altering the filtering threshold had minimal effect in our study because the number of markers was high (>6000) at all thresholds.

Complex analyses of the ASC trait data, including spatial, bivariate, and single-stage analyses, did not substantially improve the prediction accuracy over that of a simple grand mean value of the 2013 and 2015 means (Figure 8). This has some similarity to the results of GS in rye (Bernal-Vasquez et al., 2014) where complex spatial models did not improve the prediction accuracy, but using row and column effects was effective. In contrast, genomic prediction accuracy in barley was improved using a single-stage GS approach incorporating spatial effects and data from multi-environment trials (Oakey et al., 2016). The single-stage approach is considered to be the gold-standard as it fully accounts for the entire variance–covariance structure of the observed data, though it may not always be practical; for example, complicated models may fail to converge (Schulz-Streeck et al., 2013).

For a trait such as ASC which is difficult to phenotype reliably, requiring repeated field trials that are expensive, time consuming, and depend on the natural occurrence of disease that varies in prevalence and pathogen strain composition, the inclusion of genomic data can give a better estimate of the true trait value than phenotypic measurements alone. Therefore, GS is potentially valuable for pea breeding for this trait, and may be useful in different ways: to provide a better estimated BV for phenotyped lines; to predict BV for lines that are not phenotyped, and to predict BV across environments. However, for GS to be effective, the choice of training population is critical; it must adequately represent the testing population so that accurate trait values are predicted. In addition, the costs associated with genotyping must be outweighed by the cost savings generated through a reduction in phenotyping effort. GS in pea would benefit from the availability of a high quality genome sequence, and results are also likely to be improved by using a larger number of pea lines, and running more trials.

## Data Availability Statement

The mean values for the trait data in this study can be found in Supplementary File 1. The SNP data for 205 pea lines and 6019 SNPs can be found in Supplementary File 3. The raw data supporting the conclusions of this manuscript will be made available by the authors, without undue reservation, to any qualified researcher.

## Author Contributions

MC coordinated the project, made GBS libraries, did GS analyses, and drafted the manuscript. DG supervised the field trials and collection of phenotype data. CW conducted GS analyses. ST conducted bioinformatics analysis. TF collected samples for genotyping and isolated DNA. RC isolated DNA. FK collected phenotypic data. GT-V conceived and initiated the project and secured funding. All authors contributed to and approved the final manuscript.

## Conflict of Interest Statement

The authors declare that the research was conducted in the absence of any commercial or financial relationships that could be construed as a potential conflict of interest.

## Footnotes

1 http://www.deenabio.com

## Abbreviations

ASC: ascochyta blight disease score
BL: Bayesian Lasso
BRR: Bayesian ridge regression
GBLUP: genomic best linear unbiased prediction
GBS: genotyping-by-sequencing
GEBV: genomic estimated breeding value
GS: genomic selection
PFR: The New Zealand Institute of Plant and Food Research
RKHS: Bayesian Reproducing kernel Hilbert spaces regression
