# Supplementary material for: Genomic Selection for Ascochyta Blight Resistance in Pea
Source: Front Plant Sci. 2018 Dec 20;9:1878. doi: 10.3389/fpls.2018.01878 (PMC6306417; doi:10.3389/fpls.2018.01878)
Supplement: Supplementary file 4 [file Table_4.docx]

Supplementary Material

**Genomic Selection for Ascochyta Blight Resistance in Pea**

**Margaret A. Carpenter^*^, David S. Goulden, Carmel J. Woods, Susan J. Thomson, Fernand Kenel, Tonya J. Frew, Rebecca D. Cooper, Gail M. Timmerman-Vaughan**

*** Correspondence:** Margaret Carpenter: [Margaret.carpenter@plantandfood.co.nz](mailto:Margaret.carpenter@plantandfood.co.nz)

A.

B.

Supplementary file 4: Determination of population structure. (A) The mean log likelihood of K (postulated number of subpopulations) for K = 1 to15, with bars representing ± standard deviation. (B) DeltaK for 1 to 15 subpopulations as calculated by the method of Evanno et al. (2005), indicating that population structure is best represented by 2 or 10 subpopulations.
