# Supplementary material for: Genomic Selection for Ascochyta Blight Resistance in Pea
Source: Front Plant Sci. 2018 Dec 20;9:1878. doi: 10.3389/fpls.2018.01878 (PMC6306417; doi:10.3389/fpls.2018.01878)
Supplement: Supplementary file 5 [file Table_5.docx]

Supplementary Material

**Genomic Selection for Ascochyta Blight Resistance in Pea**

**Margaret A. Carpenter^*^, David S. Goulden, Carmel J. Woods, Susan J. Thomson, Fernand Kenel, Tonya J. Frew, Rebecca D. Cooper, Gail M. Timmerman-Vaughan**

*** Correspondence:** Margaret Carpenter: [Margaret.carpenter@plantandfood.co.nz](mailto:Margaret.carpenter@plantandfood.co.nz)


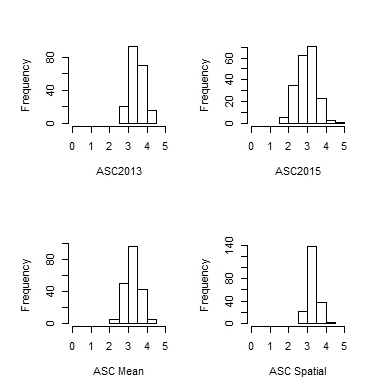


Supplementary File 5: Histograms of trait values

ASC = Ascochyta blight disease scores from trials in 2013 (ASC2013) and 2015 (ASC2015), mean values of the two trials (ASC Mean), and adjusted means from a spatial analysis (ASC Spatial).
